# CONGENITAL CYSTIC DILATATIONS OF THE BILE DUCTS: ATTEMPT FOR MODIFICATION IN CLASSIFICATION

**DOI:** 10.1590/0102-672020190004e1573

**Published:** 2021-05-14

**Authors:** Gustavo Adrian NARI, Alesio LOPEZ, Angel JOSEPH, Flavia LOPEZ, Maria Eugenia DE-ELIAS, Lorna ROMERO

**Affiliations:** 1 Hospital Tránsito Cáceres de Allende, Cirugía General, Córdoba, Cordoba, Argentina.

**Keywords:** Bile duct diseases, Caroli disease, Choledochal cyst, Doenças dos ductos biliares, Doença de Caroli, Cisto do colédoco, Imunoterapia adotiva

## INTRODUCTION

Congenital cystic dilatations of the bile duct are infrequent affecting the intrahepatic bile duct as much as the extrahepatic or both at the same time^3^ ^-^ ^4^. Vater was who first described a congenital dilatation of the extrahepatic bile duct, but was Douglas’ description in 1852 that gave full knowledge about this rare affection^17^ ^-^ ^19^. On the other hand, Jacques Caroli described in 1958 the congenital cystic dilatation of the intrahepatic bile duct as an infrequent cause of intrahepatic cholestasis^9^ ^,^ ^10^ ^,^ ^12^. These dilatations are characterized by their polymorphism as the dilatations can affect any part of the bile duct. The rareness of this pathology has caused the majority of the studies to be clinical case reports and the importance of its knowledge lies in the fact that the treatment can be wrong. 

The aim of the present communication is to present eight cases and revise the literature with the intention to evaluate if there have been changes in the therapeutic suggestions in recent years and suggest the aggregation of two subtypes in the classic classification.

## METHODS

**FIGURE 1:**
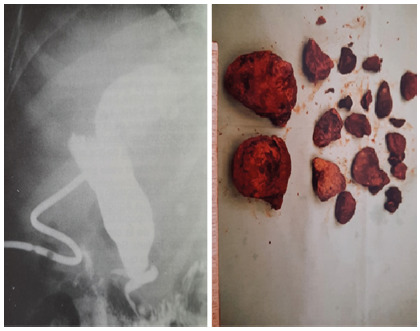
Type 4 dilatation with giant hepatolithiasis with anomalous biliopancreatic junction

In a descriptive study of a series of cases data was collected from the patients with congenital cystic dilatations of the bile duct between 1997 up to the present (23 years). For its classification we used the classification proposed by Alonso Lej with Todanis’ modification^1^ ^,^ ^13^. To classify them were used cholangiographic in pre, intra and postoperative associated to the results of the pathologic anatomy in the case of resection. Also associated with diagnostic confirmation were the indirect signs that may accompany them, such as thinning of the dilatation wall, hypoplastic gallbladder and increased duodenal-pancreatic vascular aspect, among others. The presence of the anomaly proposed by Babbit^2^ and later re-classified by the French Association of Surgery based on that of Komi et al^7^ ^,^ ^11^ was investigated (Figure 1). In patients with Caroli disease, it was specified if it was diffuse (bilobar) or unilobar; if the dilation was saccular or tubular; and if there was intrahepatic lithiasis. Caroli syndrome was considered when there was an association of congenital liver fibrosis and/or polycystic kidney disease.

## CASES REPORT

**FIGURE 2:**
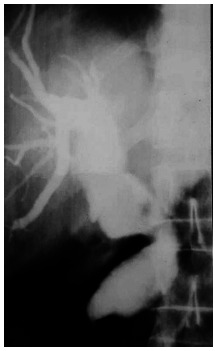
Type 1 choledocal cyst

**FIGURE 3:**
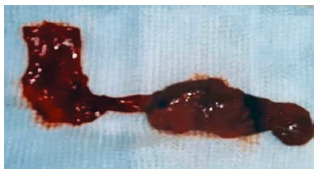
Surgical resection of gallbladder and choledocal cyst

**FIGURE 4:**
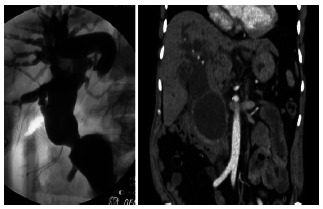
Cystic dilatation of the bile duct communicated with pancreatic pseudocyst

**FIGURE 5:**
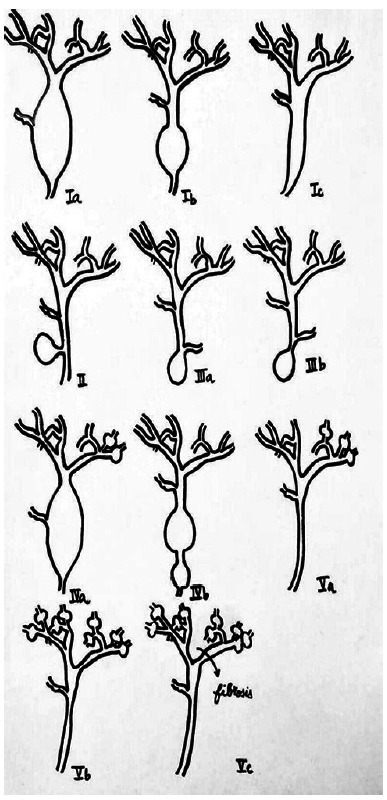
Alonso Lej-Todani classification with our modification

In Table 1 the principal data is presented of eight patients, three with Caroli disease and the other five with choledochal cysts, four were of type I and one was of type VI A. In two patients with choledochal cysts type I in which the bile duct was resected, there were anatomic changes associated to this pathology, in both there was hypoplastic gallbladder and increased duodenal-pancreatic vascular aspect. In a patient with a type 1 cyst and in the type one VI A the existence of an anomalous pancreaticobiliary junction could be objectified. As the recollection of patients was carried out in 23 years there exist different study modalities between them. In two surgical treatment was performed without preoperative knowledge of the correct diagnosis and these patients were initially approached to resolve lithiasic pathology of the bile duct. The imaging methods were of very little help in the preoperative of the patient with the choledochal cyst type VI A that operated on with a strong suspect of cholangitis. In the same way that the patient in which the transduodenal papillotomy was performed and the one who later was proposed the cystic pouch resection. The use of ursodeoxycholic acid was of great help in the patient with a hip fracture that was approached firstly by the trauma team as was the Chilean patient that had previously had a resection and had a persistent metrorrhagia hysterectomy of myomatous origin. In Figures 2 to 5 some of the most representative case images are presented. 

**TABLE 1:** Patients data

GENDER/ AGE (y)	CLINIC	LABORATORY	IMAGING	DIAGNOSTIC	TREATMENT	EVOLUTION
Male/57	Hip fracture-Jaundice-Fever and Abdominal pain	Leukocytosis-Direct bilirubin elevation and alkaline phosphatase	Ultrasound-ERCP	Diffuse tubular Caroli disease with intrahepatic lithiasis	Ursodeoxycholic acid - fracture repair	Good response. Decease because of MI in 18 days
Female/45	Uterine fibroids-Metrorrhagia-Abdominal pain-Jaundice episodes-history of left hepatic lobectomy due to Caroli disease in Chile	Alkaline phosphatase elevation-Anemia	Ultrasound-ERCP	Residual Caroli disease in segment IV. Giant Uterine Myoma	Hysterectomy - Ursodeoxycholic acid	Good evolution during 3 years and loss of control
Male/56	Abdominal pain-Fever-Dolor abdominal. Fiebre-Vomiting	Leukocytosis elevation of alkaline phosphatase	Ultrasound(acute cholecystitis)	Acute cholecystitis + Cholechochal cyst type I	Cholecystectomy and resection of VBP with Hepaticojejunostomy in Roux-en-Y	Good evolution-Symptom free during 5 years
Female/45	Choledochian syndrome-history of cholecystectomy and exploration of the bile duct one year before	Elevation of direct bilirubin and alkaline phosphatase	Ultrasound-ERCP	Choledochal cyst type I - Probable Babbit theory	Cystic pouch resection with liver - Gastrojejunostomy	Good evolution after 2 and a half years control is lost
Female/53	Abdominal pain-Fever-Choledochian syndrome	Leukocytosis-increased direct bilirubin and alkaline phosphatase	Ultrasound (Cholecystitis with dilatation of the VBP)	Cholecystitis and common bile duct lithiasis	Cholecystectomy and transduodenal papillotomy.	Good evolution. Controls through fistulography and ERCP that show type I choledochian cyst. Refuses to reoperate controlling during 3 years
Female/45	Abdominal pain-Fever-Choledochian syndrome	Leukocytosis-increased direct bilirubin and alkaline phosphatase	Ultrasound (acute cholecystitis, pulmonary vein dilation and simple liver cyst)	Acute cholecystitis, common bile duct lithiasis, liver cyst	Cholecystectomy, Choledochotomy, Left hepatectomy, extraction of multiple giant stoness - Kehr. Choledochal cyst type IV A	Good evolution - high. Anat Pathol: Gallbladder Adenoma-Hepatic cystadenoma-Clinical oncology treatment with survival of 1 year and a half
Male/51	Intermittent jaundice-Fever-Abdominal pain. History of alcoholism	Leukocytosis-increased direct bilirubin and alkaline phosphataseAPP drop	Ultrasound MRCP	Left lobar saccular Caroli dise ase. Liver cirrhosis - Child A	Left lateral sectionectomy	Good evolution. Patient dies 3 years later due to progression of alcoholic cirrhosis
Male/54	Jaundice-abdominal pain-weight loss	Leukocytosis-Increased direct bilirubin and alkaline phosphatase	Ultrasound-tomography MRCP	Type I choledochal cyst fistulized into a pancreatic pseudocyst	Cystic pouch resection and hepaticojejuno, Roux-en-Y anastomosis	Good evolution. Disappearance of pseudocyst

## DISCUSSION

Congenital cystic dilations of the bile duct in adults are rare, for Valayer cystic dilation of the extrahepatic bile duct (CHD) would occur in 1 out of 2 million births^16^; other western authors report incidence of 1 in 100,000 to 190,000 live births^4^ ^-^ ^15^; the incidence in the East would be much higher, being reported as 1 in 1,000 births in some Asian populations^3^ ^,^ ^4^ ^,^ ^19^. The female gender is more affected in the cystic dilation of the extrahepatic bile duct with a 4:1 relation, while in CD the distribution by gender is equal^4^. The majority of patients with cystic dilation of the extrahepatic bile duct are presented in childhood^16^. Yamaguchi^19^ on 1433 reports that approximately the 51% of patients were in the first decade of life and this number jumped to almost 70% in the first two decades of life, being much less frequent in adult. In comparison CD is usually diagnosed in adult.

Alonso Lej et al.^1^ made the most utilized classification and later on suffered various modifications being Flanigan´s in 1975^6^ and Todani´s in 1977^13^ the most representatives. The last one is more used and has the particularity of having incorporated, as type V, the Caroli disease. In the year 2001, we proposed a subdivision of type V in two subtypes in relation to whether it was CD or CS^10^. Today, we propose a subdivision in three subtypes as can be seen in the figure 5. This subdivision aims to standardize the therapeutic procedures that can be used. In the cystic dilation of the extrahepatic bile duct - those classified as type I - are the more frequent followed by type IV, that coincides with our patients^4^ ^,^ ^17^.

Regarding the etiology, some differences are recommended not to talk about “cystic dilation of the extrahepatic bile duct”, including them in what is called “the fibropolycystic family of liver disease”, that is, a constellation that includes other diseases^9^. Babbit^2^ proposed an anomaly in the biliopancreatic junction with two variations and with a common duct between the choledochus and the Wirsung duct longer than 10 mm round the outside of the duodenal wall. Later, other authors^7^ ^,^ ^11^ add a third more complex variety in “anse de seau” (Figure 1) and it is the most common etiological theory with a frequency of up to 90% and that justifies basically the cystic dilation of the extrahepatic bile duct type I and IV^4^ ^,^ ^15^. This theory justifies the reflux of pancreatic liquid inside the biliary tract with the consequent digestion and inflammation with later dilatation that would justify the values of amylase superior to 10000 U/L that is usually found in the bile of the dilatations^8^. Both CD and the cystic dilation of the extrahepatic bile duct type II and III have their etiology in recessive autosomal disorders that would also justify the presence of polycystic kidney disease and congenital fibrosis in CS^5^ ^,^ ^19^ ^,^ ^22^.

Regarding the clinical presentation, the classic triad of abdominal pain, jaundice and palpable tumor in cystic dilation of the extrahepatic bile duct is not present in all patients. We can say that these symptoms can occur associated with others such as fever, choluria or the presence of lithiasis. 

Our series is 23 years old and some patients were insufficiently studied. The MRCP is undoubtedly an element of great value to reach a diagnosis, while during surgery, the procedure devised by Mirizzi will also be of great help in making therapeutic decisions. The percutaneous or endoscopic approaches will not only be able to collaborate with the diagnosis, but also have the addition of being able to drain the bile duct in patients with cholangitis or hepato-cellular disorders due to cholestasis. 

One of the most important points is the malignization of the dilatation, mainly in those of type I and IV, between 2.5 % and 17.5% are reported^3^ ^,^ ^4^ ^,^ ^8^ ^,^ ^10^ ^,^ ^17^ ^,^ ^18^ ^,^ ^19^. Bruguera et al^5^ refer, that the possibility of malignancy in CD would range between 7-14%. Benjamin^4^ reports that biliary stasis favors the formation of secondary bile acids that would have mutagenic power. In our series we only had one patient with cancer, and it was from the gallbladder, the patient had an abnormality of the biliopancreatic junction and this anomaly has also been associated with the genesis of gallbladder cancer. The increased risk of developing a cholangiocarcinoma makes surgical treatment one of the first treatment options.

In what refers to the cystic dilatation of the extrahepatic bile duct, mainly in the two most frequent types, resection of the cystic bag with the performance of a Roux-en-Y hepaticojejunoanastomosis is the treatment of choice, although some authors suggest that performing a hepaticoduodenostomy would have the same results^14^; we believe that the latter favors reflux episodes of postoperative cholangitis. We have resected the dilation in three patients with good subsequent evolution, and in two we performed insufficient treatments, one of them with a multiple pathology of the bile duct that included cancer of the gallbladder and the other in which we arrived at the diagnosis postoperatively and were refused the resection. In regard to types II and III dilations, choledochele may be a recurrent cause of pancreatitis and this is the main indication for resection, whereas in the choledochian diverticulum pain or its complications would be the reasons for the resection^13^. 

Regarding type V dilatations, we propose subdivision as: Va, segmental or lobar; Vb, diffuse CD; and Vc, CS with fibrosis. This classification, in our view, is because there are different therapeutic approaches in relation to each subtype. Va type requires resectional treatment of the affected part with good results in terms of symptom control^9^ ^,^ ^10^ ^,^ ^22^. Vb type can be managed with medical treatment (ursodeoxycholic acid, antibiotics, etc) and endoscopic papillotomy^9^and in cases where control of symptoms is difficult, liver transplantation will be a weighted option. Vc type, which is associated with liver fibrosis, has indication to liver transplant, having life expectancy up to five years in 72.4%^12^.

## References

[1] Alonso-Lej F, Rever W, Pessagno D (1959). “Congenital choledochal cyst, with a report of 2 ans an analysis of 94 cases.”. Int Abstr Surg.

[2] Babbit D, Starshak R, Clemett A (1973). “Choledochal cysts: a concept of etiology.”. Am J Roentgenol Radium Ther Nucl Med.

[3] Baison G, Bonds M, Helton W, Kozarek R (2019). “Choledochal cysts: silmilarities and differences between asian and western contries.”. World J Gastroenterol.

[4] Benjamin I (2003). “Biliary cystic disease: the risk of cáncer.”. J Hepatobiliary Pancreat Surg.

[5] Bruguera M, Ros E (2006). “Enfermedad de Caroli.”. Gastroenterol Hepato.

[6] Flanigan D (1975). “Biliary cysts.”. Ann Surg.

[7] Komi N, Udaka H, Ikeda NKasiwagi Y (1977). “Congenital dilatation of the biliay tract: new classification and studywith particular reference to anomalous arrangement of the pancreaticobiliary ducts.”. Gastroenterologia Japonica.

[8] Le Roy B, Gagniere J, Filaire L, Fontarensky M, Hordenneau C, Buc E (2016). “Pancreaticobiliary maljunction and choledochal cysts: From embryogenesis to therapeutic aspects.”. Surg Radiol Anat.

[9] Moslim M, Gunasekaran G, Vogt D, Cruise M, Morris-Stiff G (2015). “Surgical management of Caroli´s disease: single center experience and review of the literatura.”. J Gastrointest Surg.

[10] Nari G, Nassar M, Moreno E, Ponce O (2001). “Congenital Cystic dilatation of the biliary tract.”. Cir Gen.

[11] Ragot E, Mabrut J, Ouaissi M, Sauvanet A, Dokmak S, Nuzzo G (2017). “Pancreaticobiliary maljunctions in european patients with bile duct cysts: results of a multicenter study of the French Surgical Association.”. World J Surg.

[12] Romine M, White J (2019). “Role of trasplant in biliary disease.”. Surg Clin N Am.

[13] Todani T, Watanabe Y, Narusue M, Tabuchi K, Okajima K (1977). “Congenital bile duct cysts: classification, operative procedures and revies of thirty-seven cases including cáncer arising from choledochal cyst.”. Am J Surg.

[14] Todani T, Watanabe Y, Toki A, Urushihara N, Sato Y “Reoperation for congenital choledochal cyst.”.

[15] Tracy L, Imagawa D (2014). “Massive congenital choledochal cyst.”. Liver Int.

[16] Valayer J, Moreaux J (1992). “Kystes de la voie biliaire.”.

[17] Visser B, Suh I, Way L, Kang S (2004). “Congenital Choledochal Cysts in adults.”. Arch Surg.

[18] Wu G, Zou S, Luo X, Wu J, Liu Z (2003). “Proliferative activity of bile from congenital choledochal cyst patients.”. World J Gastroenterol.

[19] Yamaguchi M (1980). “Congenital Choledochal cyst. Anlysis of 1433 patients in the japanese literatura.”. Am J Surg.

[20] Yonem O, Bayraktar Y (2007). “Clinical characteristics of Caroli´s disease.”. World J Gastroenterol.

[21] Yotuyanagui S (1936). “Contributions to the aethiology and pathogeny of idiopathic cystic dilatation of the common bile-duct with report of three cases: a new aethiological theory base don supossed unequal epitelial proliferation at the stage of the physiological epitelial occlusion of the primitive choledochus. Gann.

[22] Zhang D, Ji Z, Shen X, Liu H, Pan B, Dong L (2012). “Caroli´s disease: a report of 14 patients and revoew of the literature.”. Journao of Digestive Disease.

